# Dual Magnetic Particle Imaging and Akaluc Bioluminescence Imaging for Tracking Cancer Cell Metastasis

**DOI:** 10.3390/tomography9010016

**Published:** 2023-01-25

**Authors:** Ryan J. Williams, Olivia C. Sehl, Julia J. Gevaert, Shirley Liu, John J. Kelly, Paula J. Foster, John A. Ronald

**Affiliations:** 1 Department of Medical Biophysics, Schulich School of Medicine and Dentistry, Western University, London, ON N6A 3K7, Canada; 2 Robarts Research Institute, Schulich School of Medicine and Dentistry, Western University, London, ON N6A 5B7, Canada; 3 Lawson Health Research Institute, London, ON N6C 2R5, Canada

**Keywords:** magnetic particle imaging (MPI), bioluminescence imaging (BLI), cell tracking, breast cancer, reporter gene imaging, probe-based imaging, akaluc, synomag-D

## Abstract

Magnetic particle imaging (MPI) provides hotspot tracking and direct quantification of superparamagnetic iron oxide nanoparticle (SPIO)-labelled cells. Bioluminescence imaging (BLI) with the luciferase reporter gene Akaluc can provide complementary information on cell viability. Thus, we explored combining these technologies to provide a more holistic view of cancer cell fate in mice. Akaluc-expressing 4T1Br5 cells were labelled with the SPIO Synomag-D and injected into the mammary fat pads (MFP) of four nude mice. BLI was performed on days 0, 6 and 13, and MPI was performed on days 1, 8 and 14. Ex vivo histology and fluorescence microscopy of MFP and a potential metastatic site was conducted. The BLI signal in the MFP increased significantly from day 0 to day 13 (*p *< 0.05), mirroring tumor growth. The MPI signal significantly decreased from day 1 to day 14 (*p *< 0.05) due to SPIO dilution in proliferating cells. Both modalities detected secondary metastases; however, they were visualized in different anatomical regions. Akaluc BLI complemented MPI cell tracking, allowing for longitudinal measures of cell viability and sensitive detection of distant metastases at different locations. We predict this multimodal imaging approach will help to evaluate novel therapeutics and give a better understanding of metastatic mechanisms.

## 1. Introduction

At least two thirds of cancer deaths can be attributed to metastasis in vital organs such as the brain, lungs or liver [1,2]. Despite these staggering numbers and the significant amount of research devoted to this field, many of the biological mechanisms responsible for cancer metastasis remain unclear. As a result, the development of effective treatments is difficult and patient outcomes are variable [3]. Preclinical animal models are valuable in optimizing and evaluating novel treatment strategies in vivo. They allow for longitudinal studies to be carried out within an intact, living subject rather than at the cell or organ level. To enable monitoring cancer cells over time, imaging technologies have been used extensively in preclinical models to noninvasively visualize and quantify cell proliferation.

Magnetic particle imaging (MPI) is an emerging imaging technology that directly detects superparamagnetic iron oxide (SPIO) nanoparticles and is a valuable tool for in vivo tracking and quantifying SPIO-labelled cells [4,5]. The MPI system contains two opposing strong electromagnets that create quasi-static gradient magnetic fields known as the selection field. Central to this selection field is a field-free region (FFR) where there is a zero net magnetization. The FFR is scanned across the imaging field of view (FOV), and a secondary oscillating excitation field is applied to alter the magnetization of SPIOs at the FFR. The amount of SPIO present in the FFR is linearly proportional to the amount of MPI signal detected at each position and allows for calculation of the cell number, if the concentration of SPIO per cell is known [6,7,8,9,10,11].

MPI is a sensitive modality for tracking SPIO-labelled cells. The highest reported cellular sensitivity for MPI using a common SPIO, ferucarbotran, is 1000 ferucarbotran-labelled stem cells containing on average 27 pg of iron per cell (27 ng) [12]. Synomag-D [13] is a newly developed SPIO that produces more MPI signal per gram of iron and improved resolution compared to ferucarbotran [14]. Synomag-D has been used to label cells [15] and may be more optimal than ferucarbotran for MPI cell tracking. Despite its high sensitivity, there are some limitations of MPI for cell tracking. First, SPIOs can become diluted through cell division, which can result in a loss of signal over time and complicate quantification of cell number, particularly for highly proliferative cells [16]. SPIOs can be expelled from dead cells and taken up by non-transplanted cells such as macrophages, leading to an incorrect attribution of the MPI signal to the originally loaded cells [17]. Finally, MPI will produce the same signal regardless of whether the SPIO-labelled cell is dead or alive, and thus, MPI cannot be used to directly assess cell viability [17].

Bioluminescence imaging (BLI) has been widely used for preclinical cancer studies, offering a relatively inexpensive tool to evaluate cell migration, proliferation, and viability [18,19,20,21,22,23,24,25]. A luciferase reporter gene is introduced into the genome through viral or non-viral methods. When the luciferase reacts with an administered substrate, light is produced and collected by a cooled CCD camera. Since the luciferase reporter is stably expressed, BLI does not suffer from label dilution over time [26,27,28]. This makes BLI an advantageous imaging tool for longitudinal cancer studies, as highly proliferative cells can be monitored over time. Importantly, insect luciferases require ATP and oxygen as cofactors in the enzymatic reaction, resulting in a signal that is representative of live cells only. Unlike the autofluorescence that is commonly observed in fluorescence imaging, there is little endogenous bioluminescence in the body, giving BLI relatively high sensitivity for detecting engineered cells [29].

However, optical imaging is limited by the depth of penetration, since light is attenuated by biological tissue, resulting in a decreased signal at greater depths. In recent years, developments in luciferase systems have addressed some of the limitations of BLI using conventional luciferases [30]. Akaluc is a highly sensitive reporter developed through the mutagenesis of Firefly luciferase and produces light in the near-infrared region upon reaction with Akalumine [31,32,33]. Utilizing this system, researchers have demonstrated the ability to detect single Akaluc-expressing HeLa cells trapped in the lungs of mice [33]. Further, Iwano et al. were even capable of detecting an Akaluc BLI signal in the brains of freely moving marmosets, providing evidence of greatly improved sensitivity [34]. The purpose of this study was to combine MPI and Akaluc BLI for high sensitivity cancer cell tracking in a murine model of triple negative breast cancer.

## 2. Materials and Methods

### 2.1. Cell Line Origins and Culture

A derivative of 4T1 murine breast cancer cells with preferential metastasis to the brain, 4T1Br5 cells [35], were generously provided by Dr. Patricia Steeg’s lab. The 4T1Br5 cells were maintained in Dulbecco’s modified Eagle’s medium (DMEM) containing 10% fetal bovine serum (FBS) and 1% antibiotics and grown at 37 °C and 5% CO_2_.

### 2.2. Lentiviral Transduction

The 4T1Br5 cells were transduced with a lentiviral vector consisting of a pEF1α constitutive promoter driving both tdTomato (tdT) and Akaluc expression. The expression of tdT was measured by flow cytometry using a FACS Canto Cytometer (BD Biosciences, Mississauga, ON, Canada) and visualized using an EVOS FL Auto Imaging System (Thermo Fisher Scientific, London, ON, Canada).

### 2.3. Cell Characterization

The relationship between cell number and BLI signal for transduced cells was evaluated by seeding cells on a 24-well plate in 500 μL media at the following cell counts: 1 × 10^6^, 5 × 10^5^, 2.5 × 10^5^, 1.25 × 10^5^ and 6 × 10^4^ cells. Prior to imaging, 5 μL of Akalumine-HCl (5 mM TokeOni) was added directly to each well. Cells were immediately imaged on an IVIS Lumina XRMS In Vivo Imaging System (PerkinElmer, Woodbridge, ON, Canada) until maximum radiance (p/s/cm^2^/sr) was observed.

### 2.4. Cell Labeling

Cell labeling was performed with Synomag-D (Micromod Partikeltechnologie, GmbH, Hamburg, Germany), a dextran-coated nanoparticle designed for MPI consisting of iron cores ~30 nm in size and a 50 nm hydrodynamic diameter (10 mg/mL iron content). No surface functionalities were added to the Synomag-D. The 4T1Br5 cells (10^5^) were seeded in a T75 flask and grown until 80% confluency was reached. Cells were washed with PBS, and then 5 mL of serum-free DMEM media, 90 μL Synomag-D, 60 μL protamine sulphate (10 mg/mL) (Thermofisher Scientific) and 20 μL heparin (1000 U/mL) (Thermofisher Scientific) were added to culture using the protocol described [36]. After a 4 h incubation period, an additional 5 mL of DMEM with 10% FBS was added to the flask. After 24 h of incubation, cells were collected and washed three times with PBS to be used in experiments.

Perl’s Prussian Blue (PPB) staining was performed to qualitatively assess iron labeling efficiency [37]. Iron-labelled cells were imaged on an EVOS FL Auto Imaging System (Thermo Fisher Scientific, London, ON, Canada. To calculate labeling efficiency, the number of cells with visible encapsulated iron particles in three fields of view for nine different slides was counted relative to the total number of cells.

### 2.5. Cell Viability

To determine the effect of iron labeling on cell viability, labelled and unlabelled cells were stained with a 1/100 dilution of Zombie Violet (Fisher Scientific, London, ON, Canada) in PBS. Cells were then evaluated using a FACS Canto Flow Cytometer to measure fluorescence in the Zombie Violet channel (excitation nm; 450/50 nm bandpass filter). A “dead cell” control was created by incubating cells at 56 °C for 10 min prior to staining, killing approximately 20% of cells [38].

### 2.6. In Vitro BLI

To determine whether iron had any effect on BLI signal, 4 × 10^4^ labelled or unlabelled cells were added to a 24-well plate. Next, 5 μL L of Akalumine-HCL (5 mM) was added to each well prior to imaging on an IVIS Lumina XRMS In Vivo Imaging System (PerkinElmer, London, ON, Canada). This was repeated on days 1, 3, 5 and 7 post iron labeling.

### 2.7. Characterization of Synomag-D

MPI relaxometry was used to characterize the signal and resolution of Synomag-D and compared to ferucarbotran (Vivotrax, Magnetic Insight Inc., Alameda, CA, USA), a commonly used MPI tracer. Relaxometry was conducted on 3 μL of Synomag-D or Vivotrax (*n* = 3 per), using the relax module equipped on the MPI scanner. The sensitivity of both Synomag-D and Vivotrax was determined by the amplitude of the point spread function (PSF), which was normalized to the iron concentration of each SPIO, and resolution was assessed by the full width at half maximum (FWHM).

A calibration line was made to determine the relationship between iron content and MPI signal using a dilution series of known Synomag-D concentrations (50 μg, 25 μg, 12.5 μg, 6.25 μg, 3.16 μg, 1.56 μg, 0.78 μg), which were imaged individually in triplicate samples. These samples were imaged using the same parameters as the in vivo imaging (described below), and the linear relationship found between the iron mass and the MPI signal was used to quantify SPIO mass in vivo.

### 2.8. Determination of Detection Limits for Synomag-D Labelled 4T1Br5 Cells

Serial dilutions of Synomag-D-labelled breast cancer cells (1.024 × 10^6^, 5.12 × 10^5^, 2.56 × 10^5^, 1.28 × 10^5^, 6.40 × 10^4^, 3.20 × 10^4^, 1.60 × 10^4^, 8.00 × 10^3^, 4.00 × 10^3^, 2.00 × 10^3^, 1.00 × 10^3^, 5.00 × 10^2^ cells) were prepared and imaged individually in 2D using 3.0 T/m gradients, with excitation amplitudes 22 mT (X-channel) and 26 mT (Z-channel) and a 12 cm × 6 cm field of view. Three-dimensional images were acquired of lower cell numbers (8.00 × 10^3^ and below) to improve detection sensitivity. The cellular detection limit was defined as the lowest number of 4T1Br cells detected with SNR > 5 as previously described [36].

### 2.9. Animal Model

Four NU/NU nude mice were obtained from Charles River, Canada. Care was delivered in accordance with the standards of the Canadian Council on Animal Care, under an approved protocol by the Animal Use Subcommittee of Western University’s Council on Animal Care. Nude mice were used instead of immunocompetent mice to avoid any potential immunogenicity problems arising from Akaluc expression.

Those 4T1Br5 cells expressing tdT and Akaluc were cultured for one week prior to labeling with Synomag-D. One million cells suspended in 50 μL of PBS were injected into the 4th mammary fat pad of each mouse (*n* = 4) (day 0). Injections were performed under 2% isoflurane in 100% oxygen anesthesia.

### 2.10. In Vivo BLI and MPI

BLI was performed on days 0, 6 and 13 following cell injection. Immediately prior to imaging, an intraperitoneal injection of 100 μL of Akalumine-HCl (5 mM) (Sigma Aldrich, Oakville, ON, Canada) was administered. Mice were anesthetized using 1–2% isoflurane and positioned supine in an IVIS Lumina XRMS In Vivo Imaging System (PerkinElmer, London, ON, Canada). Regions of interest (ROIs) were placed over the primary tumors, and images were acquired for up to 30 min until peak radiance (p/s/cm^2^/sr) was reached for each mouse. Once the peak signal was reached, additional images were acquired with the lower half of the mouse covered with a black cloth to allow for the visualization of secondary lesions.

MPI was performed on days 1, 8 and 14 post injection. Mice were anesthetized using 1–2% isoflurane and imaged for up to 40 min. Mouse food and bedding was removed 12 h prior to imaging to minimize any unwanted gastrointestinal signal caused by ingesting iron present in mouse feed or bedding. Mice were placed in the MPI scanner headfirst in the prone position. Legs were extended and taped to the bed to spatially separate the mammary fat pad from closely situated regions, which may develop metastases. Three-dimensional isotropic MPI scans were acquired using a 3.0 T/m gradient and excitation amplitudes of 22 mT (X-channel) and 26 mT (Z-channel) with a 12 cm × 6 cm × 6 cm field of view and 35 projections.

Although imaging mice on both BLI and MPI on the same day would have garnered a better comparison of the two modalities, alternating days were chosen to avoid unnecessary stress on the mice. Previous researchers in our lab have noted that long and frequent scans requiring the administration of isoflurane resulted in high stress levels and potential early endpoints. With limited mice and veterinary services available during COVID-19, a method that minimized potential stress was mandated by the Animal Use Subcommittee of Western University’s Council on Animal Care. 

### 2.11. Image Analysis

Three-dimensional MPI images were viewed in Horos imaging software and presented as Maximum Intensity Projections (MIP). For MPI quantification, first, the background signal was determined by acquiring an image of an empty MPI sample holder, using the same imaging parameters for in vivo acquisitions. A region of interest (ROI) was manually drawn over this entire 3D image to measure the background standard deviation of image noise. A threshold of 5 times the background standard deviation (5*SD) was used to select the signal produced by the Synomag-D-labelled cells and reject noise produced by the imaging system [37]. This imaging criteria with a multiplier of 5 is based on the Rose Criteria.

The MPI signal from each ROI was calculated as the product of the mean signal (in arbitrary units) and the volume of the ROI (mm^3^). The iron mass associated with each ROI was calculated from this MPI signal and the slope of the calibration line (described earlier):

(1) $$\mathrm{Iron}\;\mathrm{Content}\;\left(\mu g\right)=\frac{\mathrm{Total}\;\mathrm{MPI}\;\mathrm{Signal}\;\left(A.U.\right)}{\mathrm{Slope}\;\mathrm{of}\;\mathrm{Calibration}\;\mathrm{Line}}$$

For BLI, images were analyzed in Living Image (PerkinElmer), and ROIs were drawn around the primary tumor signal as well as any secondary signals obtained after covering the primary tumor.

### 2.12. Ex Vivo Analysis

Mice were sacrificed with isoflurane, then perfused with 4% paraformaldehyde. The primary tumor and axillary lymph nodes were excised and stored in PBS at 4 °C degrees. Tissues were set on a plastic weighing dish, which was placed on the MPI bed. A 2D MPI scan was acquired for each excised tissue sample. The primary tumor and lymph nodes were then cut in half for two separate sectioning and staining procedures. One half of each tissue was embedded in paraffin, sectioned and stained with Perl’s Prussian Blue and Nuclear Fast Red counterstain. The other halves were cryosectioned and mounted using a mounting media containing the fluorescent nuclear dye 4′,6′-diamidino-2-phenylindole (DAPI). Slides were imaged on an ECHO Resolve microscope.

### 2.13. Statistics

All statistics were conducted using the PRISM 9 software. Student’s t-tests were used to compare data with 2 groups, whereas a one-way ANOVA was used to compare data with more than two groups. Linear regressions were used to plot data of in vitro BLI signal (radiance) or MPI signal vs cell number (*n* = 3). The goodness of fit is displayed by r^2^. Significance was determined by p-values less than 0.05.

## 3. Results

### 3.1. In Vitro Characterization of tdT-Akaluc Expressing Cells

**Figure 1:**
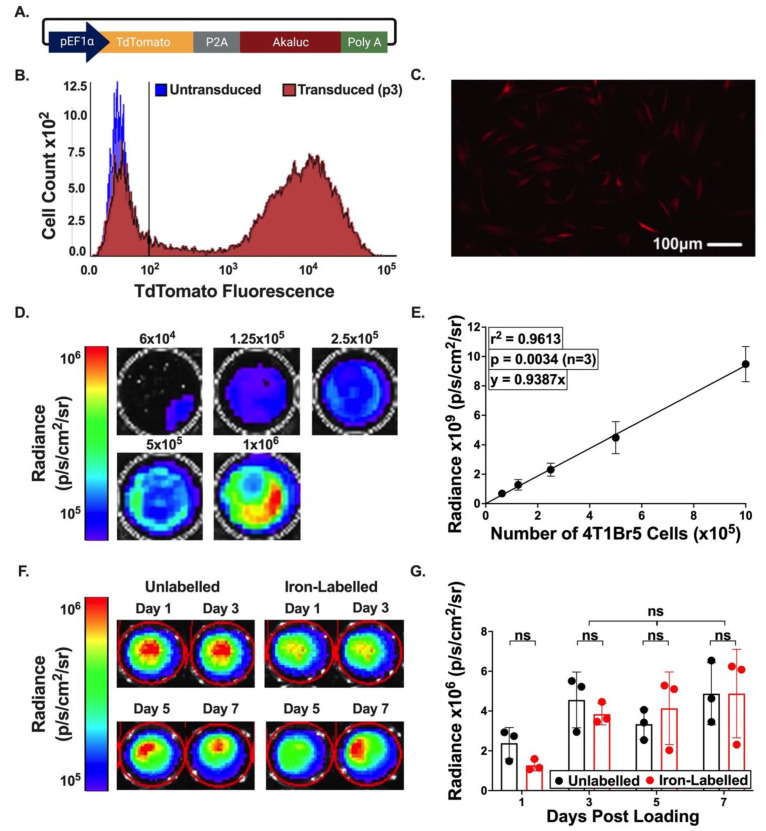
Transduction and characterization of 4T1Br5 cells. (**A**) Vector map consisting of pEF1α promoter, tdT and Akaluc. (**B**) Flow cytometry of untransduced (blue) and transduced (red) cell populations at passage 3. Cell count vs. tdT fluorescence intensity was plotted in a histogram. (**C**). Fluorescence microscopy further confirmed tdT expression. (**D**,**E**) Total BLI signal vs. cell numbers ranging from 6 × 10^4^–1 × 10^6^ cells. BLI signal scale shown in radiance (p/s/cm^2^/sr). (**F**,**G**) Total BLI signal for unlabelled (Black) and Synomag-D-labelled (Red) cells, up to 1 week post loading. ns: no significant.

A pEF1α-tdT-P2A-Akaluc expression plasmid was designed to make lentiviral vectors for stable genome integration (Figure 1A). The 4T1Br5 cells were 77.6% positive for tdT post transduction (Figure 1B), which remained constant through passages 3, 4 and 10 (76.9%, 76.3% and 79.6% tdT positive, respectfully). Fluorescence microscopy conducted on the same days showed similar percentages of cells expressing tdT fluorescence (Figure 1C).

Radiance (p/s/cm^2^/sr) measured with BLI was found to be positively and linearly correlated to the number of cells seeded (*p* = 0.0034, r^2^ = 0.9613) (Figure 1D,E). Further, labeling these TdT-Akaluc-positive cells with iron oxide (MPI tracer) resulted in no significant change (*p* < 0.001) in the produced BLI signal over a 7-day period (Figure 1F,G).

### 3.2. Characterization of Synomag-D

**Figure 2:**
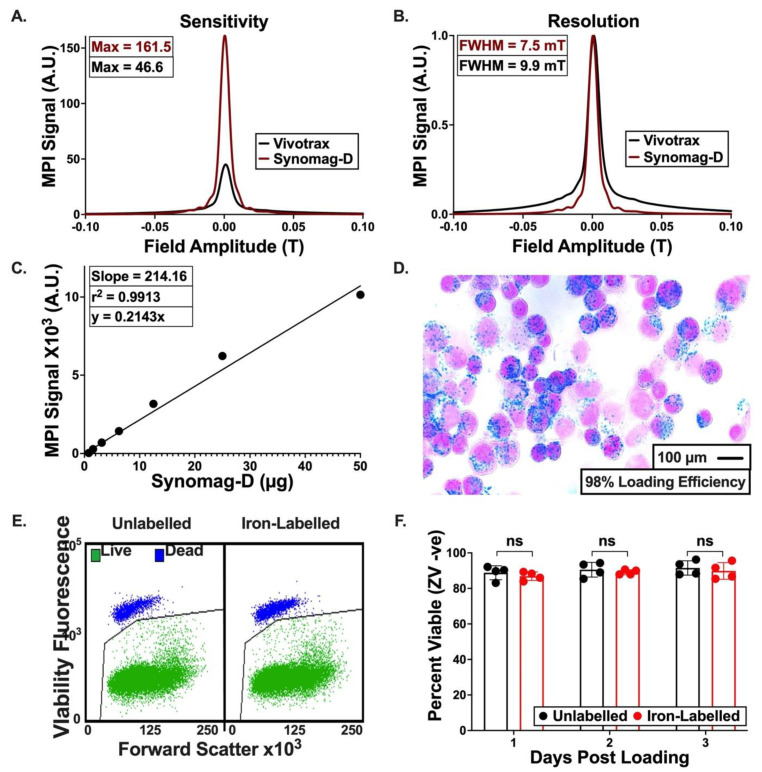
Characterization of Synomag-D. (**A**) Relaxometry curves for Synomag-D (red) and Vivotrax (black) show that Synomag-D produces 3.5 times more signal than Vivotrax, per iron mass. (**B**) Normalized relaxometry curves are used to assess signal resolution and show that the FWHM for Synomag-D is narrower than for Vivotrax. (**C**) The relationship between Synomag-D mass (ranging from 50 µg to 0.7 µg) and MPI signal produced shows a strong linear relationship. Iron content (μg) is equal to the total MPI signal (A.U.) divided by the slope of the calibration line. (**D**) Synomag-D loading of 4T1Br5-tdT-Akaluc cells was validated with Perl’s Prussian blue (PPB) stain and Nuclear Fast Red counterstain. Of these cells, 98% were PPB^+^. (**E**,**F**) Flow cytometry with the Zombie Violet viability assay shows no differences in the cell viability with Synomag-D labeling. ns: no significant.

The Relax module on the Momentum^TM^ MPI scanner was used to compare the sensitivity and resolution of Synomag-D to the more commonly used SPIO, Vivotrax. The sensitivity of Synomag-D was found to be 161.5 A.U., 3.5 times greater than the 46.6 A.U. measured for Vivotrax (Figure 2A), which was significantly different as calculated by the *t*-test (*p* < 0.0001). Figure 2B shows normalized relaxometry curves for Synomag-D and Vivotrax with FWHM values of 7.5 mT for Synomag-D and 9.9 mT for Vivotrax. For a 3.0 T/m gradient as used in this study, a resolution of 2.5 mm would be expected for Synomag-D and 3.3 mm for Vivotrax. We found a significant linear relationship between the concentration of Synomag-D and resulting MPI signal (Figure 2C), (r^2^ = 0.9913; *p* < 0.05).

### 3.3. Iron Labeling of Cells

A Perl’s Prussian Blue stain (Figure 2D) confirmed efficient cell labeling (98%), where iron appears blue within the 4T1Br5 cells. There was little extracellular iron observed. Flow cytometry was used to assess the viability of cells labelled with iron (Figure 2E,F). The percentage of cells that was viable (Zombie Violet negative) was not significantly different for labelled and unlabelled cells.

**Figure 3:**
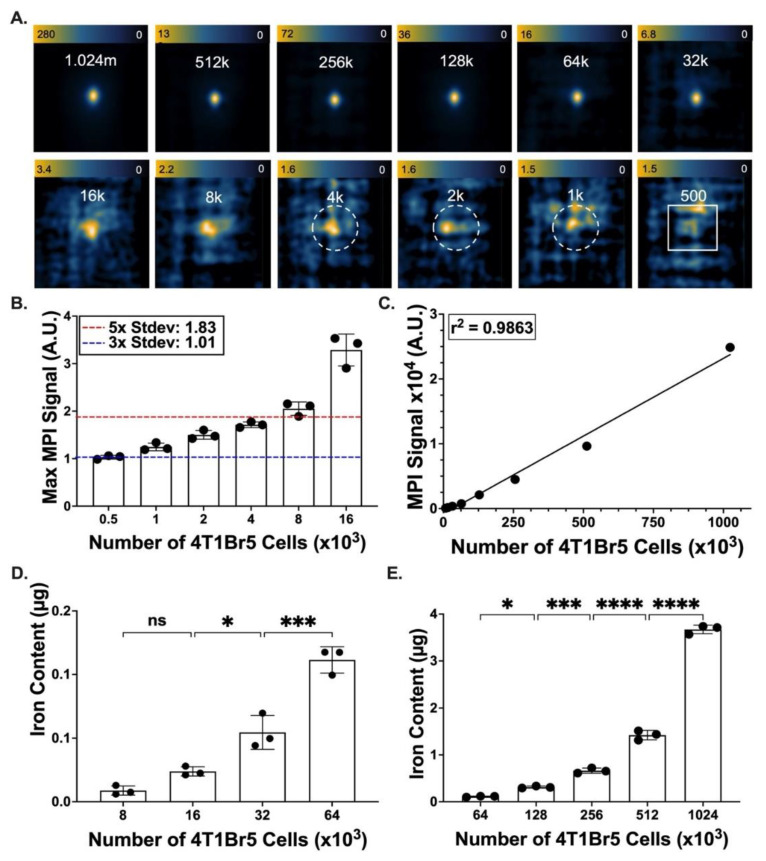
Cell detection limits for Synomag-D-labelled 4T1Br5-tdT-Akaluc cells. (**A**) 2D MPI of Synomag-D-labelled 4T1Br cells of different cell numbers (range 500–1.024 × 10^6^ cells), where “m” denotes million and “k” denotes thousand. Dotted circles indicate cell numbers that produce MPI signals with 3 < SNR < 5, and white boxes indicate cell numbers with SNR < 3. (**B**) The maximum value from the images in (**A**) are recorded for the low cell numbers (0.5–16 × 10^3^ cells). The MPI maximum values from 8000 cells exceed the threshold value of 5 × SD (red line, 1.83 A.U.). The MPI maximum values from 1000–8000 cells exceed the threshold value of 3 × SD (blue line, 1.01 A.U.). (**C**) Total MPI signal was plotted against the number of cells loaded with Synomag-D to show a positive and linear relationship. (**D**,**E**) Iron mass measured by MPI is significantly different for various cell numbers in the range of (**D**) 8–64 × 10^3^ cells and (**E**) 64–1024 × 10^3^ cells. ns: no significant. * *p* < 0.05, *** *p* < 0.001, **** *p* < 0.0001.

Different cell numbers ranged from 500 to 1.024 million Synomag-D-labelled cells (Figure 3A). The background SD of image noise was measured as 0.366 (A.U.). The maximum MPI signal in MPI images was measured for each cell number (Figure 3B, *n* = 3). Cell numbers greater than or equal to 8000 produced MPI signals that were above five times the background SD (1.83 A.U), meaning that these signals had an SNR > 5. Cell numbers greater than or equal to 1000 produced MPI signals that were above three times the background SD (1.098), meaning that signals from these cell numbers exceeded SNR > 3.

The total MPI signal (mean signal * volume) increased linearly with cell number (r^2^ = 0.9863) (Figure 3C); cell numbers below 8000 were not included in this analysis due to poor SNR. The iron mass significantly increased with cell number (Figure 3D,E). Based on the amount of iron measured from 1.024 × 10^6^ cell samples, the iron loading per cell was 3.58 pg/cell. Similarly, the Max MPI signal, being the intensity of the brightest MPI signal, also increased linearly with cell numbers greater than 8000 cells. This cannot be seen in Figure 3B, as the signal below an SNR of 5 cannot be properly assessed.

### 3.4. In Vivo Imaging of Primary Tumors

**Figure 4:**
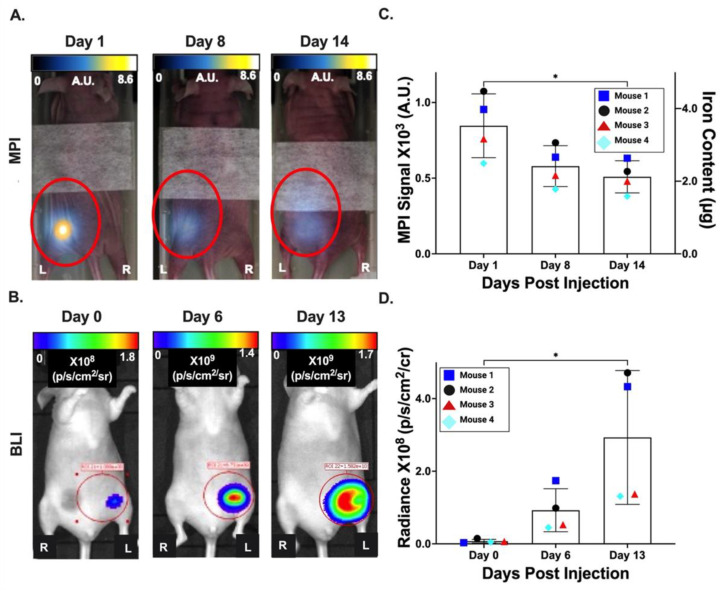
A comparison of BLI (Akaluc) and MPI (Synomag-D) detection of the primary 4T1Br5 breast tumor. (**A**) Representative in vivo MPI scans are shown for days 0, 8 and 14 post MFP injection of labelled cells. (**B**) Representative in vivo BLI scans are shown for days 1, 6 and 13 post MFP injection of labelled cells. MPI and BLI signals were co-registered with bright field images for context. Anatomical left and right are denoted by “L” and “R”. MPI signal is reported in in arbitrary units (A.U.) and enclosed by red circles. BLI signals were displayed as radiance (p/s/cm^2^/sr). (**C**) The MPI signal (left axis) and corresponding iron mass (right axis) were measured from MPI images and are reported for 4 mice. (**D**) Average BLI signal from the primary tumor was plotted for these same 4 mice. * *p* < 0.05.

Over a period of two weeks, 4T1Br5-tdT-Akaluc cells were injected into the MFP of nude mice (*n* = 4) and monitored with in vivo MPI and BLI. MPI and BLI signal co-localized with the left MFP (Figure 4A). Mice were imaged in the prone position for MPI scans and supine for BLI. The MPI signal was detected in the left MFP of all mice on days 1, 8 and 14 (508.94 ± 106.23 A.U.) (Figure 4A; left column), with a significant decrease in the signal (39%) and corresponding iron content detected between days 1 and 14 (*p* < 0.05; Figure 4B,C). The BLI signal was also detected in the left MFP of all mice on days 0 (7.31 × 10^6^ ± 1.59 × 10^6^ p/s/cm^2^/sr), 6 (9.25 × 10^7^ ± 5.92 × 10^7^ p/s/cm^2^/sr) and 13 (2.93 × 10^8^ ± 1.84 × 10^8^ p/s/cm^2^/sr) (Figure 4A right column), with a significant increase in the signal (28-fold) observed between days 0 and 13 (*p* < 0.05; Figure 4D). Supplementary Figure S1 shows the (A) MPI and (B) BLI scans for the primary tumors of the remaining three mice. This is available online at Figure S1.

### 3.5. In Vivo Imaging of Spontaneous Metastases

**Figure 5:**
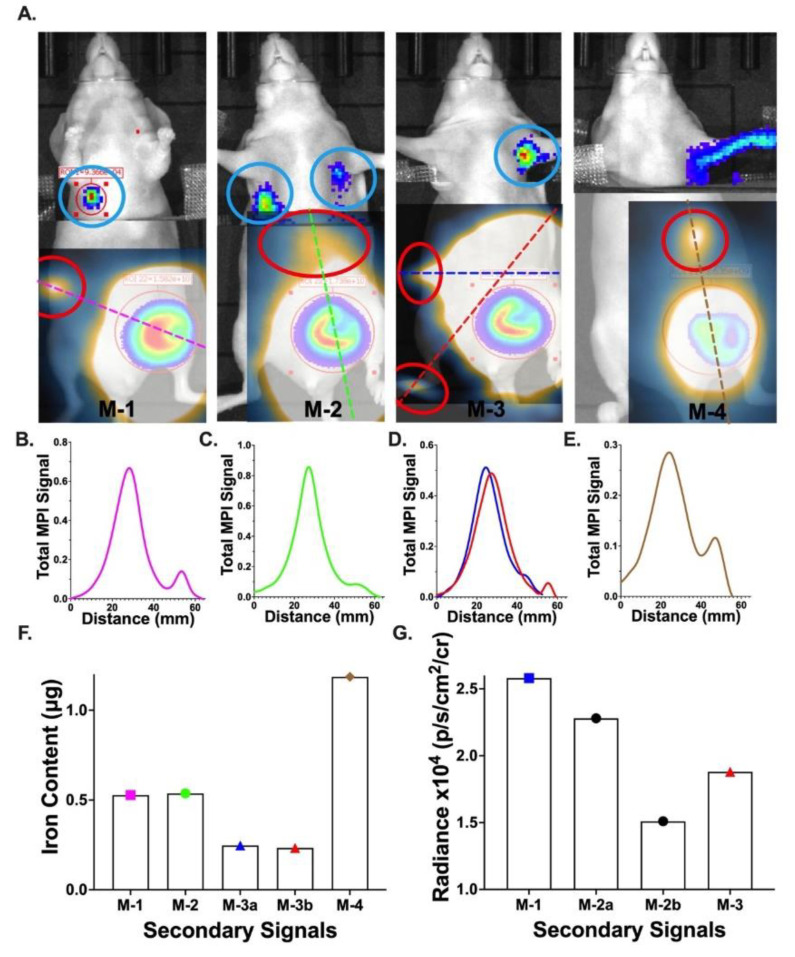
Secondary signals observed in MPI and Akaluc BLI in vivo. (**A**) Overlay of MPI, BLI and brightfield for each mouse (Mouse-1: Mouse-4). Secondary MPI signals are circled in red, and secondary BLI signals are circled in blue. (**B**–**E**) Line measurements are drawn through primary and secondary MPI signals. (**F**) Quantification of iron present in MPI secondary signals for each mouse. Mouse 3 had two secondary signals (M-3a, M-3b). Bars are color coded to the corresponding line profiles. (**G**) Quantification of secondary BLI signals from each mouse. Mouse 2 had two secondary signals (M-2a, M-2b).

On day 14, the MPI signal was detected at secondary sites throughout the body in all four mice (Figure 5A). It is probable that these signals were the result of iron-labelled metastatic cancer cells. Metastasis likely occurred between day 8 and 14, post inoculation of cells in the mammary fat pad. The locations of distant signals included the right middle (M-1, Mouse-3), the epigastric region (Mouse-2, Mouse-4), and the right footpad (Mouse-3). A line profile drawn through the MPI signal shows two distinct signal peaks for each mouse (Figure 5B–E). Quantification of the iron mass at these secondary sites is shown in Figure 5F. To visualize these secondary MPI signals, the images were displayed below 8.6 A.U. At this display level, the MPI signal from the primary tumor is saturated and extends widely from the tumor, potentially concealing any secondary MPI signals in this region. The BLI signal was also detected at secondary sites in three of four mice on day 13 by covering the lower abdomen of the animal (Figure 5A). This blocked a large portion of the signal coming from the primary tumor; thus, lower-intensity signals can be visualized using longer exposure times. Without this, the strong primary signal masks any other signals that may be present in the mice. Signals were observed in the upper right abdomen of Mouse-1, both armpits of Mouse-2 and the ipsilateral armpit of Mouse-3. We believe the signal observed for mouse 4 was due to light scatter from the primary tumor rather than the presence of Akaluc-positive cells, as the signal is dispersed over the entire arm rather than centralized to a specific region. The intensity of the BLI signal is displayed in Figure 5G. Overlaying the MPI and BLI scans allowed for a comparison of the signal location between both modalities (Figure 5A), with distinct MPI and BLI signal regions noted.

### 3.6. Ex Vivo Analysis of tdTomato Expression and Synomag D labeling in Tumors

**Figure 6:**
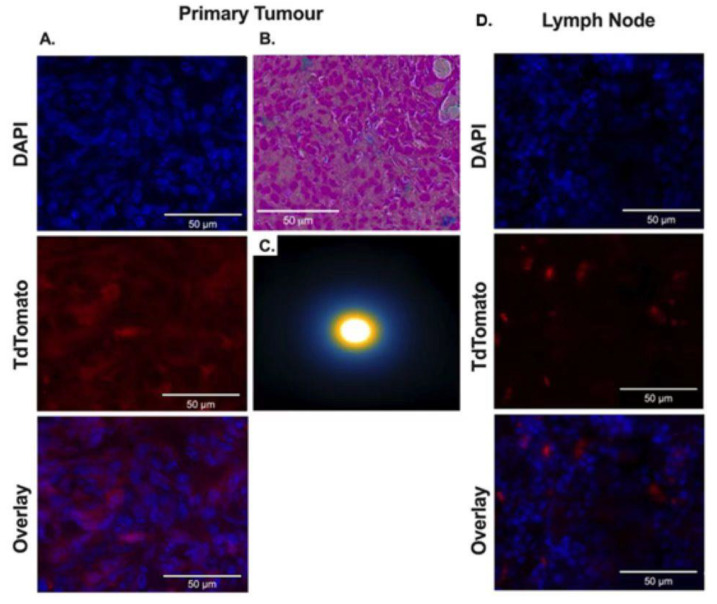
Ex vivo histology at endpoint. (**A**) Representative histology from the primary tumor and left axillary lymph node of mouse 3. DAPI (blue) stains all cell nuclei. Fluorescence in the tdTomato (red) channel confirms expression of the transduced reporter. All cells of the primary tumor (left column) were positive for tdTomato, and only a small fraction of cells in the lymph node were positive for tdTomato (right column). (**B**) PPB stain is displayed for one section of the primary tumor. Iron (blue) can be seen among the cancer cells, further confirming the presence of Synomag-D within the primary tumor. (**C**) Two-dimensional MPI of excised primary tumor highlights that Synomag-D was present in the tumor (MPI signal = 4.76 × 10^5^ A.U.).

At end points, tissues were assessed for the expression of tdT and presence of Synomag-D. The presence of tdT (red) was determined through fluorescence microscopy using a DAPI (blue) counterstain. In the primary tumor, there was a high degree of co-localization between cell nuclei (DAPI+) and expression of tdT (transplanted 4T1Br5 cells) (Figure 6A). The presence of Synomag-D in the primary tumor was further assessed ex vivo. A Perl’s Prussian-blue stain (Figure 6B) of sectioned tissues confirmed the presence of iron in the primary tumor. MPI scans of the primary tumor (Figure 6C) produced a signal indicating iron presence. tdT-expressing cells were also identified in the left axillary lymph node (Figure 6B), suggesting metastasis of these cells, and this corresponds to the location of the in vivo BLI signal (Figure 5A). Neither the MPI scan nor the PPB showed any iron in the lymph node ex vivo, consistent with our in vivo imaging results.

## 4. Discussion

With the ever-increasing prevalence of metastatic cancer deaths [3], there is an urgent need for highly sensitive imaging technologies that can noninvasively visualize and quantify tumor burden in preclinical cancer models. Our lab, among others, previously used iron-based cellular MRI to track the fate and biodistribution of cancer cells in vivo [39,40,41,42]; however, the indirect detection of SPIO through signal blooming makes quantifying cell numbers difficult [43]. Alternatively, MPI, a new player in the field of cell tracking, can directly detect SPIO-labelled cells, allowing for quantitative hotspot imaging [4,5,6], although the sensitivity is not yet on par with cellular MRI, and in vivo resolution has been an ongoing problem. BLI has often been paired with MRI or MPI due to its ability to reliably track both proliferation and viability, something not possible with probe-based modalities. BLI has traditionally been limited by its low depth penetration; however, the development of Akaluc and Akalumine, a near-infrared BLI reporter system, greatly mitigates this. Akaluc BLI has been shown to offer lower signal attenuation, superior depth penetration, and higher sensitivity for small animal imaging compared to the traditional luciferase reporter systems [31,32,33].

This is the first study to combine MPI and Akaluc BLI for quantitative, high-sensitivity tracking of metastatic cancer cells in a preclinical breast cancer model. We believe the implementation of this strategy will be extremely valuable in studying the underlying mechanisms of cancer metastasis as well as provide the imaging framework to evaluate therapeutic efficacy in vivo more accurately during the development of new anti-cancer agents.

Combining MPI with optical imaging techniques such as BLI can help overcome some of the limitations of MPI. Yu et al. became the first group to visualize cancer cells in vivo with MPI when they tracked the uptake of IV-administered MPI nanoparticles by a subcutaneous breast cancer tumor via the enhanced permeability and retention effect (EPR) [10]. BLI was used on day 1 to confirm cell location. In 2018, both Song et al. and Jung et al. tracked cancer cells labelled with MPI nanoparticles conjugated to fluorescent moieties. However, by using fluorescent moieties, rather than a fluorescent reporter gene, this method did not allow for the visualization of proliferation or viability [44,45]. Parkins et al. in 2020 tracked circulating tumor cells with MPI; however, no optical imaging system was used [46]. Later, in 2020, Melo et al. used MPI to track the metastasis of cancer cells to the brains of mice [47]. In 2021, Knier et al. tracked patient-derived xenografts with both MPI and BLI; however the same mice were not imaged on both modalities [48]. In 2021, Makela et al. used MPI and Red-Fluc BLI to track 4T1 cell implantation, tumor growth and the subsequent spontaneous metastasis to the lymph node. However, the BLI signal was not quantified [49]. Red-Fluc from the Italian firefly *Luciola italica* is a red-shifted luciferase with an emission maximum of 614 nm at pH 6.0 [50]. Based on the emission spectra of Red-Fluc and Akaluc BLI (maxima of 677 nm with Akalumine-HCl) [32], Akaluc should have far superior depth penetration and sensitivity in vivo.

Though the literature on the implementation of Akaluc BLI for cancer cell tracking is sparse, this system has demonstrated the ability to sensitively track both tumor burden and metastasis, although it has yet to be paired with MPI. In 2018, Iwano et al. were able to detect single Akaluc-expressing cells implanted in the lungs of mice, mimicking metastasis to one of the deepest regions [33]. In 2021, both Liu et al. and Ichise et al. tracked spontaneous lung metastases in vivo using Akaluc BLI [51,52]. Additionally, the treatment of these metastases by NK cells was evaluated in the Ichise paper; however, at the time of writing, this has yet to be peer reviewed [52].

Here, for the first time, we were able to detect and quantify cancer cells in the same mouse using MPI and Akaluc BLI. There was high concordance between the location of MPI and BLI signal at early time points, indicating that the same cell populations were being imaged on both modalities. Additionally, there was no observable background signal in the gut for either MPI or Akaluc BLI. A reoccurring problem in MPI is a gut signal caused by contamination of food and bedding [53,54]; however, by fasting as well as the removal of bedding prior to imaging, this was mitigated. Removing this background signal allows for the detection of cells in regions that may have otherwise been hidden, such as in the lower abdomen. Additionally, several sources have reported a background liver signal with Akalumine in the absence of Akaluc [10,45]; however, this was not observed for this experiment.

By the experimental endpoint, the MPI and BLI signal differed greatly. The MPI signal decreased by roughly 40% by day 14. Highly proliferative cells will dilute the iron tracer upon cell division. The dispersion of these cells over a larger area, or potentially to secondary sites in the form of metastases, may account for some of the decrease in signal. Additionally, a large parentage of the change is likely due to clearance of the Synomag-D. Scavenger cells such as macrophages are known to take up free iron particles for clearance in the liver and spleen. This free iron can be the result of cell death, or it may come from the active expulsion of the iron by live cells. Interestingly, this drop in signal occurred at a far more rapid rate than that which was previously reported by Song et al. in 2018, in which the MPI signal only decreased by 20% over a 20-day span [45]. This increased rate in signal loss may suggest that for highly proliferative cell lines, MPI may not be a reliable tool for the long-term quantification of cells. In contrast, the BLI signal observed on day 13 was more than 28-fold greater than the signal detected on day 1, which was expected, as the reporter gene is passed on to daughter cells during tumor growth. These inversed trends between MPI and BLI data match the results reported by Knier et al. and Makela et al. in 2021 and demonstrate that BLI can provide information on cell proliferation for future MPI studies [48,49].

Additionally, by the experimental endpoint, both MPI and BLI were able to detect a signal from regions outside of the primary MFP tumor, indicating secondary metastases. Both BLI and MPI have previously demonstrated the ability to detect metastases in mice [33,47,49,51]; however, in this study, MPI was able to detect iron in regions that were covered up during our BLI metastases imaging protocol. Specifically, Akaluc BLI was able to detect signals in distal regions such as the armpit or upper gut; however, doing so required covering the primary tumor. An MPI signal was not detected in these distant lesions. This was likely due to the dilution of tracer minimizing the amount of iron present within the cells that seeded the metastasis. The ex vivo histology supports this theory, as no iron was detected in a lymph node that was positive for a BLI signal and negative for a MPI signal in vivo. However, a MPI signal was detectable in more proximal regions to the primary tumors, as no covering was needed. Line plots through the primary tumor and the secondary lesion revealed two distinct signal peaks, indicating that this secondary signal was not associated with the primary tumor. The fact that a MPI signal was detected in distant metastases suggests that circulating tumor cell clusters, or tumor emboli, may be involved, since a single iron-loaded cancer cell would have to proliferate to form a metastasis, and in doing so, the iron would be diluted between progeny, limiting detection by MPI [55]. We have observed something similar for lymph node metastasis detected with MRI [56]. The discrepancy between secondary locations observed through MPI and BLI highlights the benefits of using both modalities to give a more complete picture of cancer cell fate in this spontaneous metastasis model. The use of a single modality may result in missing critical information, hindering the true assessment of disease progression.

While the sensitivity of Akaluc as a BLI reporter gene has been well established, it is important to also consider the added sensitivity of Synomag-D for MPI cell tracking. In concurrence with Vogel et al. 2021 and Sehl et al. 2020, we found that Synomag-D was nearly four times as sensitive as the standard Vivotrax, with a similar resolution for detecting particles in vitro [6,14]. For cellular detection limits, it is important to consider the type of SPIO, the labeling efficiency (pg of iron/cell) and the imaging acquisition parameters. For example, cells labelled with the same amount of iron but with two different SPIOs may have significantly different MPI signals. Some cells take up much more iron than others; for example, stem cells, which are much larger, take up more iron than cancer cells. In this study, we could detect and quantify 8000 Synomag-D-labelled breast cancer cells in vitro, which contained 3.58 pg iron/cell (29 ng iron). In other in vitro experiments performed in our lab, we detected as few as 2000 Synomag-D labelled dendritic cells that contained 3.5 pg iron/cell (7 ng iron) [54], 4000 ferucarbotran-labelled MSC that contained 19 pg iron/cell (76 ng iron) and as few as 8000 ferucarbotran-labelled breast cancer cells that contained 9 pg iron/cell (72 ng iron) [53]. Zheng et al. reported the detection of 1000 cells; these were embryonic stem cells labelled with 27 pg iron/cell (27 ng) [12]. These values highlight the need to consider all factors when making statements about cell detection limits. MPI has not yet reached the single cell sensitivity that is possible with MRI or Akaluc BLI; however, advancements in tailored MPI tracers and imaging techniques will soon allow for the detection of smaller metastases at earlier timepoints. Additionally, our study provides evidence that labeling cells with Synomag-D has no significant effects on cell viability or BLI signal. These findings are encouraging for the potential clinical use of Synomag-D as an MPI tracer for additional studies.

MPI studies are often limited by the dilution of MPI tracer over time, as well as bystander cell labeling [17,39,40,41,57]. By adding complementary Akaluc-BLI to our MPI study, this was no longer a concern; however, there are still a few limitations to be considered. First, though the in vivo results are promising, a small sample size was used. Future studies should be conducted to assess the reproducibility of these findings as well as to explore other imaging time points. Second, due to the potential stress of isoflurane anaesthesia, MPI and BLI were performed on different days, which limited the comparison that could be made between the two modalities. This is mandated at our facility. Additionally, mice were imaged in the prone position for MPI and the supine position for BLI. Images needed to be flipped horizontally before the signals could be aligned. Quantification of MPI signals provides its own challenges. Though the primary and secondary signal locations were distinct from one another, the tails of each signal overlapped to some degree, as seen in the associated line plots (Figure 5B–E). As a result, the exact quantification of secondary lesions is difficult. Imaging mice in the same orientation will be explored in future experiments.

Though combining MPI with Akaluc BLI helps to minimize some of the limitations of each modality, there are still limitations inherent to each modality that could not be overcome. As BLI relies on the detection of emitted light by reporter genes, signal is limited by depth. Even with a NIR reporter such as Akaluc BLI, which reduces the effect of tissue attenuation, BLI can only be used to detect cells within a few centimeters of the surface. For imaging more deeply situated regions, this means BLI can only be used for small animals, reducing its clinical relevance. Fluorescent imaging, a different optical imaging modality that also relies on detected light, has found clinical use as a scope for surgery. Using BLI for similar clinical applications with the methods described in this manuscript is highly improbable. The requirement for implanting genetically modified cells into humans comes with incredible and possibly unforeseeable risk, in addition to ethical concerns. Reporter gene imaging typically has higher specificity than other imaging modalities; however, it is not without false positives. As BLI cannot distinguish between ATP produced by the animal and ATP produced by microorganisms, bacterial contamination can increase the levels of the BLI signal observed. A sterile technique and proper sanitation can mitigate this; however, several researchers have shown that common sanitizers and detergents used in cleaning equipment have a small impact on ATP-based bioluminescence measurements. Additionally, the diet of each animal may have an impact on the BLI signal, as food present in the gut may cause a BLI signal. All mice in this experiment were fed the same diet but could have eaten different quantities. In the future, mice could be fasted prior to BLI, as done with MPI.

In this study, we demonstrated for the first time the ability to track metastatic cancer cells in vivo with both MPI and Akaluc BLI. As highlighted by the differences in secondary metastasis detection, MPI and BLI provide complementary information on cell fate, which will provide a more complete picture on the intricacies of cancer metastasis. In this study, breast cancer cells are the first and only cell type of any kind to be tracked with this multimodal imaging approach. Future experiments will use this framework to track both proliferative and non-proliferative cell populations, including other cancers, stem cells and immune cells. Specifically, we hope to use this model to track the delivery and therapeutic effect of cellular therapies such as CAR-T or NK cells, the former of which has already begun separately with both modalities [58,59]. Additionally, in the future, MPI may be combined with other reporter gene modalities that are clinically relevant, such as PET.

## Data Availability

The data presented in this study are available on request from the corresponding author.

## Supplementary Materials

The following are available online at https://www.mdpi.com/article/10.3390/tomography9010016/s1, Figure S1: A comparison of BLI (Akaluc) and MPI (Synomag-D) detection of the primary 4T1Br5 breast tumor for all mice.
