# Supplementary material for: Dual Magnetic Particle Imaging and Akaluc Bioluminescence Imaging for Tracking Cancer Cell Metastasis
Source: Tomography. 2023 Jan 25;9(1):178–94. doi: 10.3390/tomography9010016 (PMC9968184; doi:10.3390/tomography9010016)
Supplement: Supplementary file 1 [file tomography-09-00016-s001.zip › tomography-2135387-supplementary.pdf]

Supp 1.

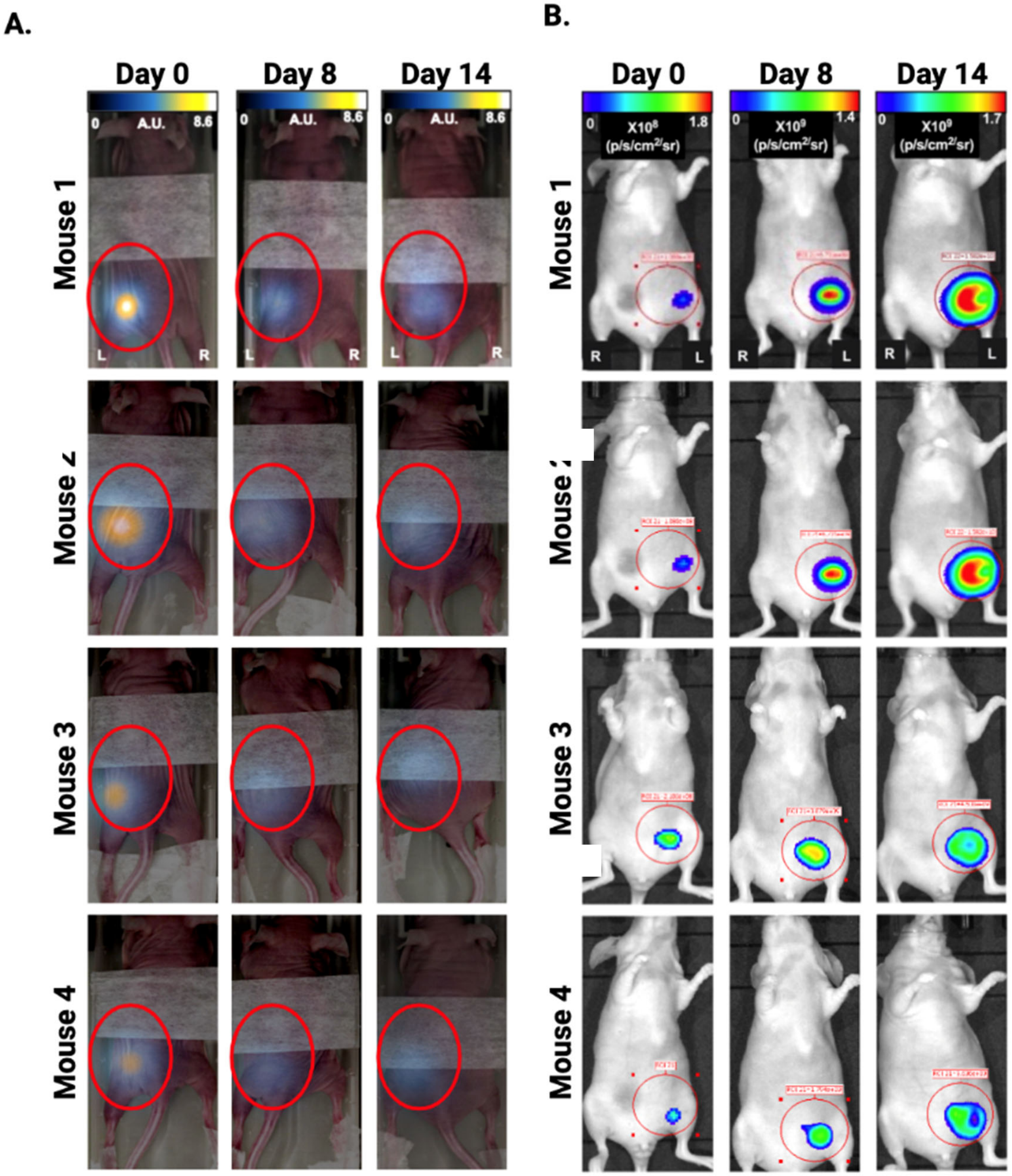

Supplementary Figure S1. A comparison of BLI (Akaluc) and MPI (Synomag-D) detection of the primary 4T1Br5 breast tumor for all mice. cells. a. *In vivo* MPI scans are shown for days 0, 8, and 14 post MFP injection of labelled cells (n=4). **b.** *In vivo* BLI scans are shown for days 1, 6, and 13 post MFP injection of labeled cells (n=4). MPI and BLI signals were co-registered with bright field images for context. Anatomical left and right are denoted by "L" and "R". MPI signal is reported in in arbitrary units (A.U.) and enclosed by red circles. BLI signals were displayed as radiance ($p/s/cm^2/sr$). All MPI scans are in the same orientation, with the same scale. All BLI images are in the same orientation with the same scale.
